# Bone marrow-derived monocytes give rise to self-renewing and fully differentiated Kupffer cells

**DOI:** 10.1038/ncomms10321

**Published:** 2016-01-27

**Authors:** Charlotte L. Scott, Fang Zheng, Patrick De Baetselier, Liesbet Martens, Yvan Saeys, Sofie De Prijck, Saskia Lippens, Chloé Abels, Steve Schoonooghe, Geert Raes, Nick Devoogdt, Bart N. Lambrecht, Alain Beschin, Martin Guilliams

**Affiliations:** 1 Unit of Immunoregulation and Mucosal Immunology, VIB Inflammation Research Center, Ghent 9000, Belgium; 2 Department of Biomedical Molecular Biology, Ghent University, Ghent 9000, Belgium; 3 Myeloid Cell Immunology, VIB, Brussels 1050, Belgium; 4 Cellular and Molecular Immunology Research Group, Vrije Universiteit Brussel, Brussels 1050, Belgium; 5 Department of Internal Medicine, Ghent University, Ghent 9000, Belgium; 6 VIB Bio Imaging Core, Ghent 9000, Belgium; 7 Microscopy Core Facility, VIB, Inflammation Research Center, Ghent 9000, Belgium; 8 In Vivo Cellular and Molecular Imaging Research Group, Vrije Universiteit Brussel, Brussels 1050, Belgium

## Abstract

Self-renewing tissue-resident macrophages are thought to be exclusively derived from embryonic progenitors. However, whether circulating monocytes can also give rise to such macrophages has not been formally investigated. Here we use a new model of diphtheria toxin-mediated depletion of liver-resident Kupffer cells to generate niche availability and show that circulating monocytes engraft in the liver, gradually adopt the transcriptional profile of their depleted counterparts and become long-lived self-renewing cells. Underlining the physiological relevance of our findings, circulating monocytes also contribute to the expanding pool of macrophages in the liver shortly after birth, when macrophage niches become available during normal organ growth. Thus, like embryonic precursors, monocytes can and do give rise to self-renewing tissue-resident macrophages if the niche is available to them.

## Abstract (web summary)

Tissue-resident macrophages are mostly derived from embryonic progenitors. Scott *et al*. develop a mouse model to specifically deplete Kupffer cells (KC) *in vivo* and show that monocyte-derived cells can repopulate KC niche and behave similar to their embryonically-derived counterparts.

## Graphical abstract

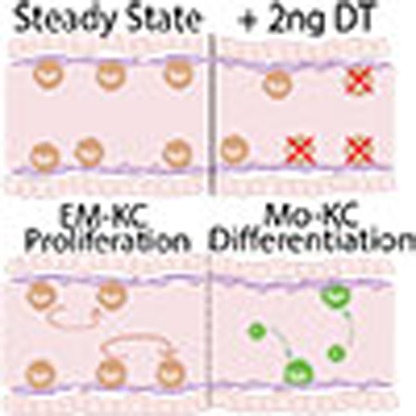

Tissue-resident macrophages (mφs) are found in all organs of the body, where they are adapted to perform specific functions required for tissue homeostasis^1^. Contrary to most immune cells which derive from hematopoietic stem cells, tissue-resident mφs develop prenatally from embryonic progenitors, including yolk-sac mφs and foetal liver monocytes^2^,^3^,^4^,^5^,^6^,^7^,^8^,^9^,^10^. The specific contributions of these two types of embryonic progenitors differs between tissue-resident mφ populations^2^,^3^,^6^,^8^,^9^,^10^,^11^. Crucially, mφs of embryonic origin, irrespective of progenitor type, have the capacity to self-renew and hence in most tissues the mφ niche remains populated by these embryonically derived mφs in adulthood, with no input from circulating monocytes^2^,^3^,^6^,^7^,^8^,^12^. Indeed monocytes only seem to contribute to the tissue-resident mφ niche following lethal irradiation and under these circumstances the monocyte-derived mφs are quite distinct from their embryonic counterparts^13^,^14^. These new insights have undermined the concept of the common mononuclear phagocyte system, where the circulating monocyte was seen as the central progenitor of all tissue mφs (ref. 15). However, circulating monocytes do contribute to the macrophage pool in the intestine and the heart. In these tissues, mφs of embryonic origin are replaced by circulating monocytes after birth and these monocyte-derived mφs are subsequently continuously replenished by the same monocyte progenitors throughout life^4^,^10^. These recent studies have lead to the dogma that circulating monocytes cannot generate self-renewing tissue-resident mφs (ref. 2). However, it may also be that these tissues are not permissive to mφ self-renewal irrespective of their origin and hence the validity of this dogma remains unclear. To examine if circulating monocytes have the capacity to generate self-renewing mφs we sought to create space in a mφ niche where self-maintenance occurs. Here, we demonstrate that circulating monocytes can generate self-renewing *bona fide* Kupffer cells (KCs), the resident mφs in the liver.

## Results

### Identification of *Clec4f* as a KC-specific gene

**Figure 1:**
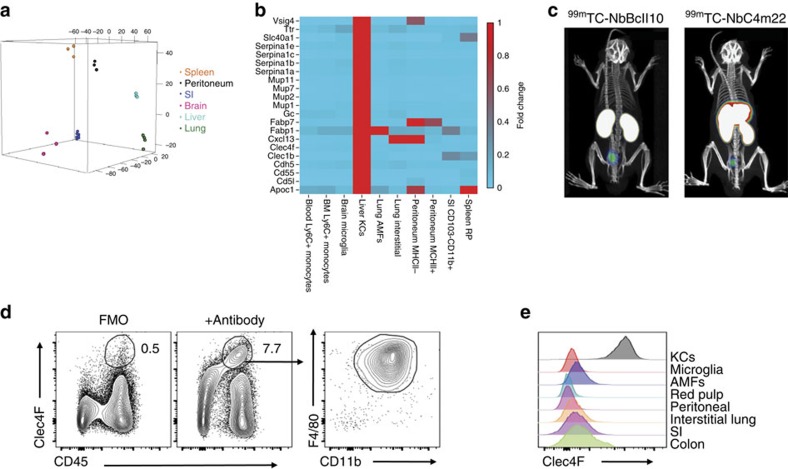
Identification of a KC-specific gene. (**a**) Principle component analysis of transcriptional profiles of KCs compared with other tissue-resident mφs. (**b**) Heatmap of mean fold change in core KC genes. Expression level in KCs was set at one. (**c**) SPECT/*μ*CT images 1 h post injection with control (NbBcII10) or anti-Clec4f (NbC4m22) ^99m^Technetium-labelled nanobodies. Representative of two experiments with *n*=6. (**d**) Clec4F, CD45, F4/80 and CD11b expression in liver cells. Representative of 5 experiments. (**e**) Clec4F expression by mφ populations. AMFs, alveolar mφs; red pulp, splenic red pulp mφs; SI, small intestinal Mφs; Representative of two experiments.

To date, it has been impossible to selectively deplete only one population of tissue-resident mφs, without disturbing the entire mononuclear phagocyte system. Thus we first sought to generate an *in vivo* model in which the liver KC niche could be selectively emptied. To this end, we identified unique KC genes, by comparing KCs to other tissue-resident mφs previously arrayed by the Immgen Consortium^16^ (Fig. 1a,b). Analysis of the tissue-specific expression of these genes using BioGPS revealed *Clec4f* to be liver-specific (Supplementary Fig. 1). This in combination with the fact that Clec4F has been previously described as a KC-specific marker^13^,^17^, led us to follow-up on this C-type lectin. To validate this, we generated and administered Technetium-99 labelled Clec4F-specific nanobodies to mice and performed whole-body imaging. The Clec4F-specific-binding signal was restricted to the liver (Fig. 1c), while non-specific signals were observed in the kidney and bladder, the normal pathway of nanobody (Nb) excretion. Flow cytometric analysis of whole liver homogenates confirmed that Clec4F was a KC-specific marker, with all Clec4F^+^ cells having the CD45^+^F4/80^+^CD11b^int^ phenotype of KCs (Fig. 1d), while mφs in other tissues did not express Clec4F (Fig. 1e).

### Generation and validation of a KC-DTR mouse model

**Figure 2:**
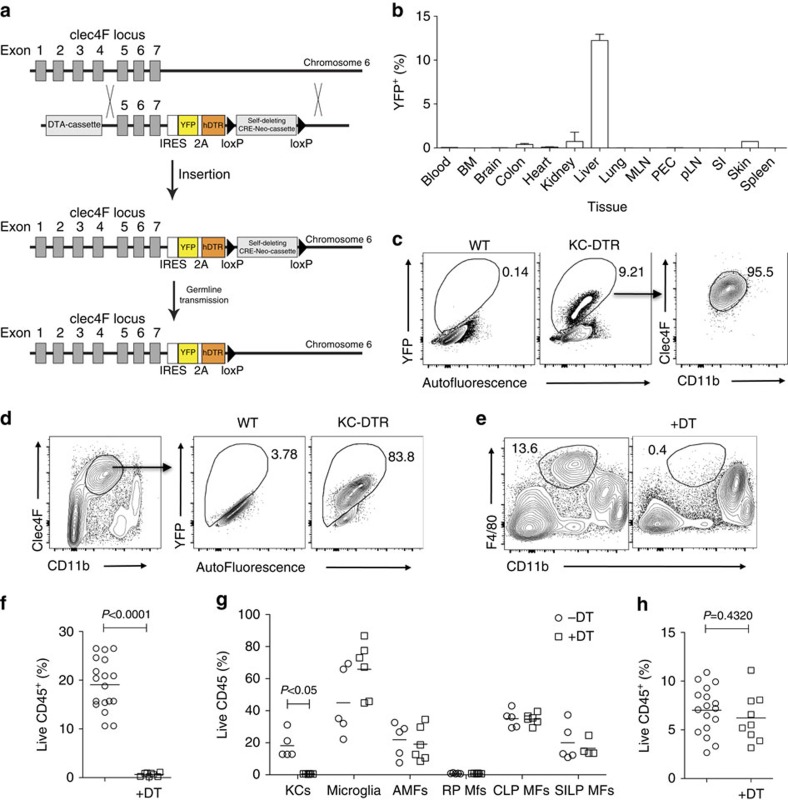
Generation and validation of KC-specific KC-DTR mice. (**a**) Schematic showing generation of KC-DTR mice. (**b**) Percentage of YFP-expressing cells amongst live cells in different tissues of KC-DTR mice. One experiment. *n*=3 per group. Error bars represent s.e.m. (**c**) Liver homogenates from KC-DTR mice or WT littermate controls were examined for YFP expression and YFP^+^ cells were examined for Clec4F and CD11b expression. Representative of three experiments. (**d**) YFP expression by Clec4F^+^CD11b^int^ KCs from KC-DTR mice or WT littermate controls. Representative of three experiments. (**e**) F4/80 and CD11b expression amongst live CD45^+^ cells 24 h post administration of DT to KC-DTR mice or WT littermate controls. Representative of five experiments. (**f**) Proportion of KCs amongst live CD45^+^ cells 24 h post administration of DT. Data are pooled from three experiments. *n*=18 (controls) or 8 (+DT). *P*<0.0001 two-way Student's *t*-test. (**g**) Tissue-resident mφs (AMFs, Alveolar mφs; CLP MFs, Colonic Lamina Propria mφs; RP MFs, splenic red pulp mφs; SILP MFs, Small intestinal Lamina Propria mφs) as percentage of Live CD45^+^ cells 18 h post DT administration to KC-DTR mice or WT littermate controls. Data are pooled from two experiments. *n*=5 for all groups except SILP Mφs +DT where *n*=4. *P*<0.05 two-way analysis of variance (ANOVA) with Bonferroni post-test. (**h**) Neutrophils and eosinophils as percentage of live CD45^+^ cells 24 h post administration of DT (+DT) compared with controls. Data are pooled from three experiments. *n*=17 (control) or 9 (+DT). *P*=0.4320 two-way Student's *t*-test.

We next generated a mouse in which KCs expressed the human diphtheria toxin receptor (KC-DTR mice) by introducing an expression cassette encoding for an internal ribosome entry site, a yellow fluorescent protein (YFP), a self-cleaving 2A peptide and the human DTR into the 3′ untranslated region of the *Clec4f* gene (Fig. 2a). YFP expression confirmed specific labelling of KCs in KC-DTR mice (Fig. 2b–d) and administration of diphtheria toxin (DT) resulted in 100% ablation of F4/80^+^CD11b^int^ KCs within 24 h (Fig. 2e,f). All other tissue-resident mφs were left intact after systemic DT administration (Fig. 2g and Supplementary Fig. 2). Importantly, DT-mediated loss of KCs did not result in overt inflammation in the liver, as there was no eosinophil or neutrophil infiltration (Fig. 2h and Supplementary Fig. 3), and mice appeared healthy.

### Depleted embryonic KCs are replaced by monocyte-derived KCs

**Figure 3:**
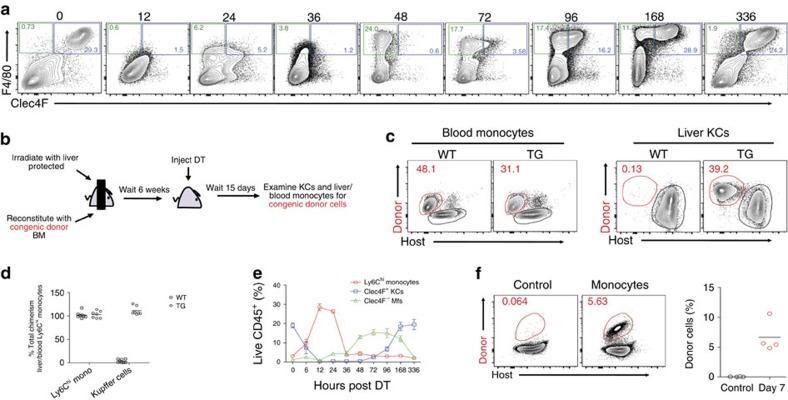
Depleted em-KCs are rapidly replaced by mo-KCs. (**a**) Expression of Clec4F and F4/80 at indicated time-points (h) post administration of DT. FACS-plots are pre-gated on Live CD45^+^SiglecF^−^Ly6G^−^Ly6C^−^ single cells. Data are representative of three experiments. (**b**) Schematic for generation of shielded chimeras to test origin of repopulating Clec4F^+^ mφs. (**c**,**d**) KC-DTR (TG) or WT littermate control (WT) shielded chimeras were administered DT and blood Ly6C^hi^ monocytes (live CD45^+^CD11b^+^Ly6G^−^Ly6C^hi^) and liver Clec4F^+^ mφs (live CD45^+^Ly6C^−^Ly6G^−^SiglecF^−^F4/80^+^Clec4F^+^) were examined for the proportion of congenic donor BM-derived cells. Percentage of total chimerism amongst liver Ly6C^hi^ monocytes and Clec4F^+^ KCs is shown. Chimerism amongst blood Ly6C^hi^ monocytes was set at 100%. Data are pooled from three experiments. *n*=8 (WT) or 7 (Tg). (**e**) Proportion of Ly6C^hi^ monocytes, Clec4F^+^ KCs and Clec4F^−^ mφs in the liver as a percentage of live CD45^+^ cells at various time-points post DT administration. Data are pooled from three experiments. *n*=18 (0h), 6 (6, 36h), 10 (12, 96h), 8 (24, 48, 72h), 12 (168h) or 7 (336h). Error bars represent s.e.m. (**f**) 8 × 10^5^–10 × 10^5^ BM Ly6C^hi^ monocytes (live CD45^+^CD11b^+^Ly6G^−^Ly6C^hi^CD115^+^) from WT congenic mice were transferred into DT-administered KC-DTRxCCR2^−/−^ recipients and 7 days later, live CD45^+^Clec4F^+^F4/80^+^CD11b^int^ KCs in the liver were examined for the presence of congenic donor cells. Data are pooled from two experiments. *n*=4 per group.

Using this model, we next investigated the consequences of completely emptying the KC niche. Within a period of 4 (96 h)–14 days (336 h) of DT administration, a population of Clec4F^+^F4/80^+^ cells resembling KCs gradually appeared in the liver (Fig. 3a,e and Supplementary Fig. 4). These arose through an F4/80^+^Clec4F^−^ intermediate, which could be seen in the liver already 48 h after DT administration (Fig. 3a,e). To examine the origin of the repopulating liver mφs, we generated shielded bone marrow (BM) chimeras in which KC-DTR mice were irradiated with their abdomen shielded to prevent genotoxic radiation damage and inflammation in the liver. In addition, abdomens were shielded from irradiation to prevent any confounding effects of irradiation on the origins of the KC population. These were then reconstituted with congenic wild-type BM (Fig. 3b). Administration of DT depleted KCs in these mice and these were subsequently repopulated at a chimerism level equal to that of blood Ly6C^hi^ monocytes (Fig. 3c,d), demonstrating that the repopulating KCs were of BM origin. As the initial stages of KC repopulation following DT depletion were associated with a transient increase in Ly6C^hi^ monocytes in the liver (Fig. 3e), we examined directly whether the repopulating KCs were derived from Ly6C^hi^ monocytes. Thus we adoptively transferred CD45.1^+^ BM Ly6C^hi^ monocytes (Supplementary Fig. 5a) into CCR2^−/−^xKC-DTR mice which had received DT to deplete the endogenous KCs. This confirmed that Ly6C^hi^ monocytes act as progenitors of the repopulating KCs (Fig. 3f). Thus, hereafter we refer to these as monocyte-derived KCs (mo-KCs).

### Mo-KCs compete with em-KCs for KC niche repopulation

**Figure 4:**
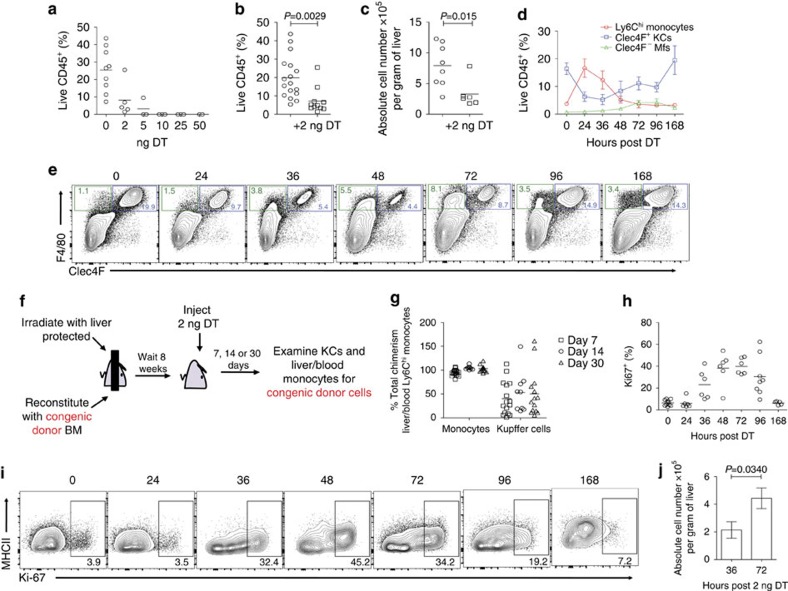
Mo-KCs compete with em-KCs for KC niche repopulation after partial depletion. (**a**) Percentage of KCs among live CD45^+^ cells 24 h following administration of various doses of DT. Data are from one experiment. *n*=9 (0 ng), 5 (2 ng), 3 (5, 10, 25 ng) or 2 (50 ng). (**b**) Percentage of KCs among live CD45^+^ cells in KC-DTR or WT littermate controls 24 h post administration of 2 ng DT. Data are pooled from 3 experiments. *n*=17 (control) or 11 (+2 ng DT). *P*=0.0029 two-way Student's *t*-test. (**c**) Absolute number of KCs per gram of liver tissue in KC-DTR or WT littermates 24 h post administration of 2 ng DT. Data are pooled from two experiments. *n*=8 (controls) or 6 (+2 ng DT). *P*=0.015 two-way Student's *t*-test. (**d**) Proportion of Ly6C^hi^ monocytes, Clec4F^+^ KCs and Clec4F^−^ mφs in the liver as a percentage of live CD45^+^ cells at various time-points post administration of 2 ng DT. Data are pooled from two experiments. *n*=11 (0 h), 6 (24, 36, 48, 72 h), 8 (96 h) or 5 (168 h). Error bars represent s.e.m. (**e**) Clec4F and F4/80 expression on total Live CD45^+^Ly6C^−^Ly6G^−^SiglecF^−^ cells from KC-DTR or WT littermate that received 2 ng DT at indicated time-points (h). (**f**) Schematic of shielded chimeras to determine origin of Clec4F^+^ KCs following partial KC depletion. (**g**) Percentage of total chimerism amongst Ly6C^hi^ monocytes and KCs in mice treated with 2 ng DT 7, 14 and 30 days earlier. Chimerism amongst blood Ly6C^hi^ monocytes was set at 100%. Data are pooled from three experiments. *n*=16 (d7), 9 (d14) or 14 (d30). (**h**) Percentage of Ki-67^+^ Clec4F^+^ KCs at indicated time-points post administration of 2 ng DT. Data are pooled from two experiments. *n*=11 (0 h), 6 (24, 36, 72 h), 8 (96 h) and 5 (168 h). (**i**) Ki-67 and MHCII expression on Clec4F^+^ KCs at indicated time-points (h) post administration of 2 ng DT. (**j**) Absolute number of KCs per gram of liver 36 and 72 h post partial depletion with 2 ng DT. Data are pooled from two experiments. *n*=6 per group. *P*=0.0370 two-way Student's *t*-test. Error bars represent s.e.m.

These results suggest that availability of an empty KC niche, as achieved by depletion of embryonic KCs (em-KCs), allows circulating monocytes to differentiate into mo-KCs. To determine if recruited monocytes were able to compete with em-KCs, we next titrated the dose of DT to only partially deplete the liver niche of KCs (Fig. 4a–c). Treatment with 2 ng of DT depleted ∼80% of em-KCs and similarly to full-depletion triggered a transient appearance of Ly6C^hi^ monocytes followed by F4/80^+^Clec4F^−^ mφs and the subsequent restoration of the full F4/80^+^Clec4F^+^ KC population (Fig. 4d,e). To examine whether the recruited monocytes were responsible for this repopulation, we generated shielded chimeras, reconstituted them with congenic wild-type BM and then administered the partial dose of DT (Fig. 4f). Under these conditions, the level of chimerism averaged only 50% of that of blood Ly6C^hi^ monocytes (Fig. 4g), despite ∼80% of resident em-KCs having been depleted. Thus monocyte differentiation could not account for the complete repopulation of the KC niche when a proportion of endogenous em-KCs remained. We thus hypothesized that proliferation of the remaining em-KCs may also play a role in the repopulation of the KC niche. This was confirmed in two ways. First, we found increased proportions of Clec4F^+^ KCs expressing the proliferation marker Ki-67 between 36 and 96 h after DT administration (Fig. 4h,i). Second, we measured an increased number of F4/80^+^Clec4F^+^ KCs between 36 and 72 h (Fig. 4j). This expanded population of F4/80^+^Clec4F^+^ KCs at 72 h can only be of embryonic origin as Clec4F^+^ mo-KCs are not observed until 96 h post depletion (Fig. 3a,e). Thus, following partial depletion, monocytes compete with proliferating resident em-KCs for niche occupancy.

### Mo-KCs are highly homologous to em-KCs

**Figure 5:**
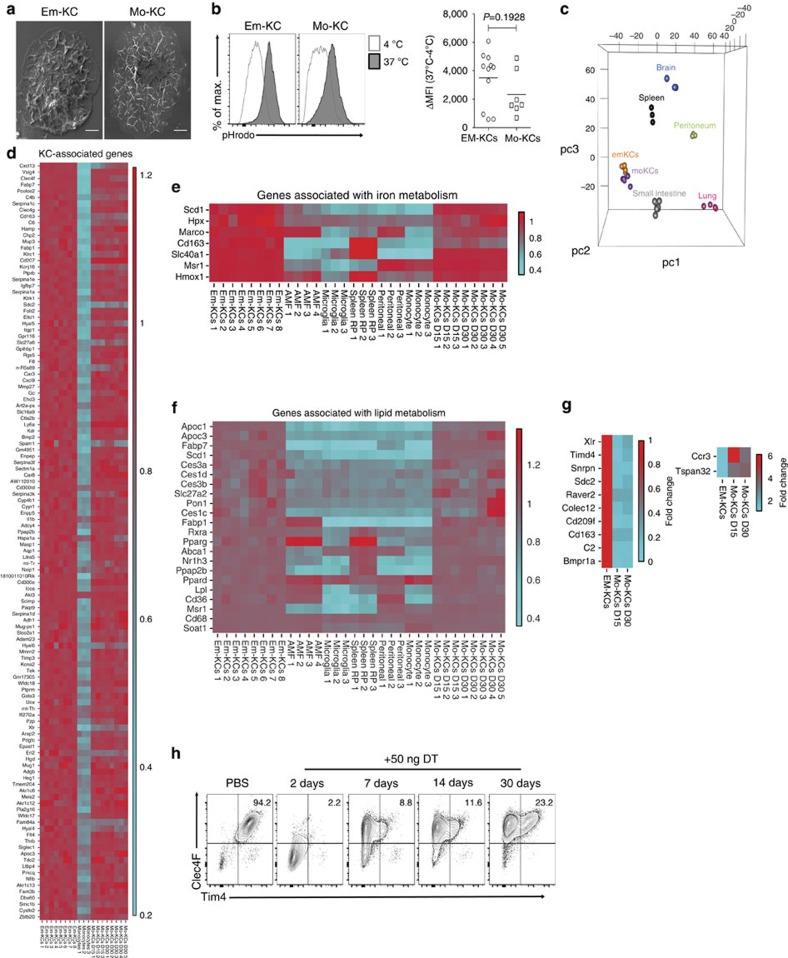
Mo-KCs are highly homologous to em-KCs. (**a**) SEM imaging of em-KCs and mo-KCs 30 days post DT administration. Scale bar, 4 μm. Images are representative of *n*=3 per group. (**b**) Uptake of *E. coli* bioparticles conjugated to the pH-sensitive fluorescent pHrodo dye by em-KCs and day 7 mo-KCs shown as Δ mean fluorescence intensity at 37–4 °C (control). Data are pooled from two experiments. *n*=7 (mo-KCs) or 11 (em-KCs). *P*=0.1928 Two-way Student's *t*-test. (**c**) Principle component analysis of em-KCs, mo-KCs (pooled day 15 and day 30 post DT) sorted as shown in Supplementary Fig. 5b and other tissue-resident mφ populations sorted by the Immgen Consortium. (**d**–**g**) Heatmap of fold change in expression of (**d**) top 100 genes enriched in Em-KCs when compared with all other tissue-resident mφ populations and blood Ly6C^hi^ MHCII^−^ monocytes, which were sorted and arrayed by the Immgen Consortium (**e**) core iron metabolism genes enriched in em-KCs (**f**) core lipid metabolism genes enriched in em-KCs and (**g**) all genes differentially expressed by at least 1.5-fold between em-KCs and mo-KCs at day 30. In all heatmaps, mean expression of each gene by em-KCs was set at one. (**h**) Clec4F and Tim4 expression by live CD45^+^CD11b^+^Ly6C^−^F4/80^+^ liver cells measured at 2, 7, 14 and 30 days post DT administration. Data are representative of 1–3 experiments at the various time-points with *n*=2–4 per group.

Using full-body genotoxic irradiation to deplete em-KCs, others found that hematopoietic cells can give rise to liver mφs, but the resulting cells acquired <50% of the tissue-specific enhancers of their em-KC counterparts^13^. This could suggest that a substantial component of the KC-specific gene signature may reflect embryonic origin, thus, we investigated whether our mo-KCs adopted the characteristic features of em-KCs. Scanning electron microscopy (SEM) of FACS-purified em-KCs and mo-KCs showed the two cell-types to be morphologically similar, being large, granular and with an adherent appearance (Fig. 5a). In addition, mo-KCs and em-KCs were equally capable of phagocytozing pHRodo-labelled *Escherichia coli* bioparticles (Fig. 5b). Finally, we performed microarray analysis on mo-KCs obtained 15 and 30 days post depletion (Supplementary Fig. 5b); comparing their transcriptional profiles with em-KCs. Principle component analysis (PCA) showed that mo-KCs and em-KCs clustered together and separately from the other tissue mφs (Fig. 5c). The mo-KCs expressed the 100 most specific em-KC-associated genes identified through comparison of em-KCs with other tissue-resident mφ populations (Fig. 5d). Mo-KCs also expressed similar levels of the genes associated with iron (Fig. 5e) and lipid (Fig. 5f) metabolism, two putative em-KC functions. Consistent with the high degree of genetic homology between mo-KCs and em-KCs, we found only 54 genes to be differentially expressed, of which merely 12 were >1.5-fold different, with the greatest mean difference being only 3.5-fold (*Cd209f;* Fig. 5g). Interestingly, day 30 mo-KCs were even closer to the em-KC profile than day 15 mo-KCs (Fig. 5g), suggesting that most genes would be equally expressed with further time after replenishment. To validate these differences at the protein level, we stained for Tim4 (a receptor for eat-me signals of apoptotic cells and damaged red blood cells, encoded by *Timd4*). Fitting with the temporal increase in *Timd4* mRNA expression, mo-KCs were found to gradually acquire Tim4 protein with time (Fig. 5h).

### Mo-KCs acquire the capacity to self-renew

**Figure 6:**
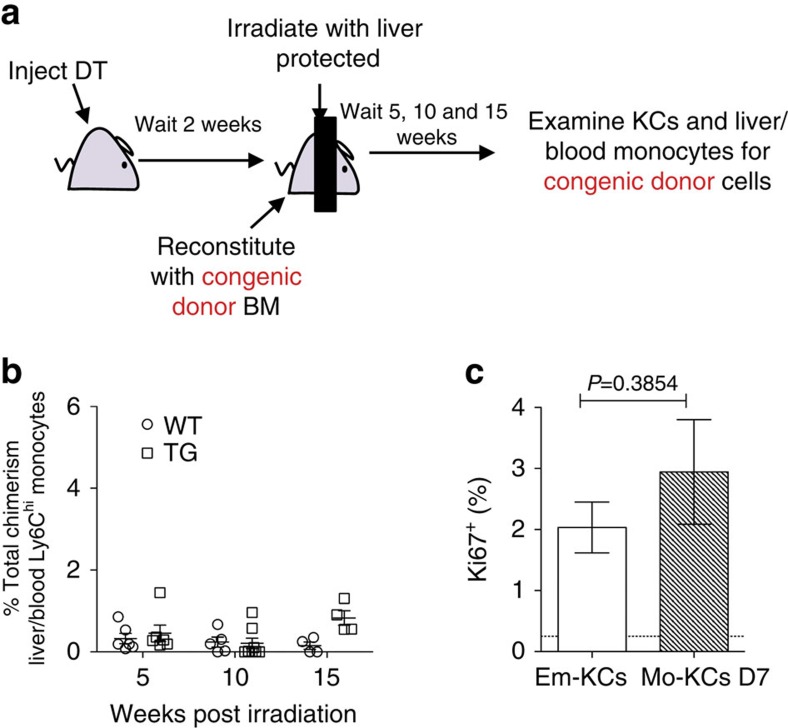
Mo-KCs acquire the capacity to self-renew. (**a**) Schematic of shielded chimeras to determine lifespan of mo-KCs. (**b**) Percentage of total chimerism amongst KCs in fully depleted (50 ng DT) KC-DTR (TG) and WT littermate controls. Chimerism amongst blood Ly6C^hi^ monocytes was set at 100%. Data are pooled from two experiments. *n*=6 (WT and TG 5weeks), 5 (WT 10 weeks), 8 (TG 10 weeks) or 4 (WT and TG 15 weeks). (**c**) Percentage of Ki-67^+^ em-KCs or mo-KCs at day 7 post administration of DT. Data are pooled from two experiments. *n*=6(em-KCs) and 7(mo-KCs). Dotted line represents FMO. *P*=0.3854, two-way Student's *t*-test. Error bars represent s.e.m.

Although it is currently believed that the property of self-maintenance is restricted to embryonic mφ progenitors^2^,^18^,^19^,^20^, the kinetics of KC repopulation (Figs 3e and 4d) suggested that monocytes may colonize the empty KC niche in a single wave and then self-renew. To explore this directly, we allowed the liver to be colonized by mo-KCs for 15 days after KC depletion, before subjecting the mice to shielded irradiation and reconstitution with congenic wild-type (WT) BM (Fig. 6a). Under these conditions, we could not observe KCs derived from newly recruited BM progenitors in the liver 5, 10 or 15 weeks after irradiation (Fig. 6b). Thus, the mo-KC population self-maintains for at least 4 months without any additional input from circulating monocytes. In support, we found that mo-KCs have the same degree of Ki-67 expression as unmanipulated em-KCs (Fig. 6c), suggesting they have equal rates of proliferation and self-renewal. These results demonstrate that once circulating monocytes gain access to an artificially emptied niche, they acquire the capacity to self-renew within 2 weeks.

### Mo-KCs are generated in the first weeks of life

**Figure 7:**
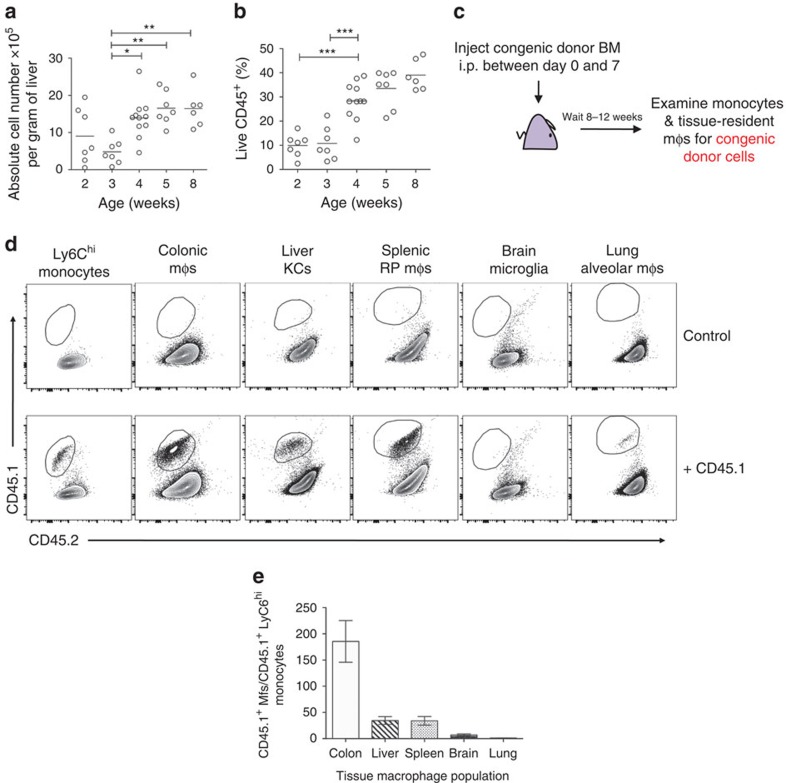
Mo-KCs are generated during normal liver growth in the first weeks of life. (**a**) Absolute number of KCs per gram of liver at indicated time-points after birth. Data are pooled from two experiments. *n*=7 (2, 3 ,5 weeks), 11 (4 weeks) or 6 (8 weeks). *P<0.05, **P<0.01. One way analysis of variance (ANOVA) with Bonferroni post-test. (**b**) Percentage of KCs among live CD45^+^ cells at indicated time-points after birth. Data are pooled from two experiments. *n*=7 (2, 3, 5 weeks), 11 (4 weeks) or 6 (8 weeks). ***P<0.001. One way ANOVA with Bonferroni post-test. (**c**) Schematic of adoptive transfer of congenic total BM to newborn pups to assess monocyte contribution to KC pool during growth. Mice were given a single injection of 8 × 10^6^ BM cells within the first 7 days after birth. (**d**) Representative FACS plots showing engraftment of congenic donor BM into liver Ly6C^hi^ monocytes, colonic macrophages, liver KCs, splenic red pulp macrophages, brain microglia and lung alveolar macrophages, gated as shown in Supplementary Fig. 2. (**e**) Ratio (%) of macrophages derived from CD45.1 congenic BM and Ly6C^hi^ monocytes derived from CD45.1 congenic BM 8–12 weeks post adoptive transfer (i.p.) of CD45.1 congenic BM to CD45.2 WT mice in the colon, liver, spleen, brain and lung. Data are pooled from five experiments. *n*=23. Error bars represent s.e.m.

We next sought to investigate if monocytes also give rise to KCs under more physiological conditions of mφ niche availability. We hypothesized that normal postnatal organ development might represent such a situation. Liver mass increases exponentially due to hepatocyte proliferation between birth and week 4 (ref. 21), and liver sinusoids are only properly fenestrated postnatally^22^. Liver growth was associated with an increase in the proportion (% of live CD45^+^ cells) and total number of KCs during the first weeks after birth, stabilizing at 4–5 weeks of age (Fig. 7a,b). To examine monocyte contribution during this period of development, we performed a single adoptive transfer of congenic BM into unmanipulated WT pups within the first 7 days after birth (Fig. 7c). Engrafted transplanted cells were identified 8–12 weeks later amongst the pool of KCs (Fig. 7d,e). Interestingly, splenic red pulp mφs and colonic mφs were also partially derived from transplanted cells, whereas alveolar mφs and microglia were not (Fig. 7d,e). These data demonstrate that circulating BM-derived monocytes are not only capable of repopulating the KC niche following artificial DT-mediated depletion but also contribute significantly to the KC pool in the growing liver after birth.

## Discussion

It was recently proposed that only mφs of embryonic origin have the capacity to self-renew^2^ and that genetic engineering of monocytes would be necessary to generate self-repopulating mφs *in vivo*^2^,^18^,^19^,^20^. However, we show here that BM-derived monocytes give rise to self-renewing KCs when the niche is rendered available. In addition to the unexpected ability of the BM mo-KCs to self-renew, mo-KCs show a significant phenotypical and transcriptional overlap with their embryonic counterparts. Indeed only 12 genes were ⩾1.5-fold differentially expressed between em- and mo-KCs, with some of these differences appearing to be only temporal. Importantly, availability of the KC niche to BM-monocytes is not an artifact of experimental KC depletion, but also occurs during the first weeks of life in unmanipulated mice, yielding a significant fraction of mo-KCs in the adult KC pool. Although these data seem to contrast with recent reports employing fate-mapping, which proposed that most resident mφs derive exclusively from embryonic precursors^3^,^5^,^6^,^7^,^12^, it is important to note that these previous studies were performed either in prenatal or adult mice, and thus will have missed the postnatal period of organ development. Two recent studies employing fate-mapping in the embryo in fact found a declining proportion of labelled mφs as mice reached adulthood^4^,^8^, and although this was unexplained in these studies, based on our findings we propose that this is due to dilution of the mφ pool by BM-derived monocytes during organ growth.

Integrating our data with previous work, we propose a model in which embryonic progenitors colonize the tissues before birth^2^,^3^,^4^,^5^,^6^,^7^,^8^, but as the mouse grows, BM-derived monocytes fill up additional mφ niches that become available, competing with the resident populations. This scenario occurs in the liver and spleen, but not in the brain or lung. Therefore, we propose to also incorporate niche accessibility in the model to explain the differing contributions of the distinct mφ progenitors to the tissue-resident mφ pools in various organs^23^. Accordingly, microglia in the brain arise exclusively from yolk-sac mφs as the niche is available and accessible to these progenitors while the blood–brain barrier later prevents foetal liver monocytes or BM-monocytes from accessing the brain mφ niche^8^,^12^. Conversely, alveolar mφs derive predominantly from foetal liver monocytes as the alveolar space is not formed and hence not accessible when yolk-sac mφs predominate in the embryo^6^,^12^. After birth, the lung epithelium impedes the influx of BM monocytes^23^, resulting in the exclusive foetal monocyte origin of alveolar mφs^2^,^6^,^8^. In addition, strong, but as of yet unknown, quorum sensing mechanisms appear to be at play, such that once mφ niches are full, there is no further input from circulating progenitors even in mφ niches that are accessible to BM-monocytes like the spleen and the liver. However, in the heart or intestine, the scenario of niche filling keeps repeating itself, and gradually all em-mφs are replaced^4^,^10^. In the intestine, niche availability is created in part by signals from the microbiome that likely compromise mφ lifespan^4^,^23^, whereas in the heart it might be mechanical damage causing mφ demise^24^. However, how monocytes are recruited to these tissues on niche availability remains an open question. IL-33 has been proposed to be involved during Listeria infection in the liver^25^ but whether this holds true under homeostatic conditions or in other tissues remains to be seen. Finally, although we show here that repopulation of the KC pool upon partial DT-mediated depletion occurs through the simultaneous proliferation of the remaining em-mφs and the differentiation of monocytes into mo-KCs, it appears that inflammation and tissue damage can influence the competition between these two repopulation mechanisms. Indeed, repopulation of the KC pool after *N*-acetyl-p-aminophenol overdose was shown to be solely mediated by em-KC proliferation^26^, while KC repopulation following Listeria monocytogenes was mainly driven by differentiation of monocytes into mo-KCs^25^. While these two studies appear contradictory, during Listeria infection, the remaining em-KCs did not proliferate^25^ perhaps explaining why mo-KCs were generated in this model compared with *N*-acetyl-p-aminophenol treatment. Thus, identifying the signals regulating em-KC proliferation and monocyte recruitment and differentiation in response to niche availability will be crucial in understanding and manipulating this process and hence future research should be focused on understanding the factors that control quorum sensing and lifespan in specific macrophage niches and how this is influenced by disease.

## Methods

### Mice

KC-DTR (CD45.1 and CD45.2), KC-DTRxCCR2^−/−^, wild-type (CD45.1, CD45.1/CD45.2 and CD45.2) C57Bl/6 (Harlan Olac) mice were maintained under specific-pathogen free conditions at the animal house of the VIB/UGent Inflammation Research Center. All mice were backcrossed for at least five generations onto the C57Bl/6 background and were used between 6 and 12 weeks of age unless otherwise stated. Male and female mice were used. All experiments were carried out in accordance with the ethical committee Ghent University—Faculty of Science/VIB.

### Construction of the KC-DTR mice

The Clec4F-specific knock-in mouse B6-Clec4f^tm1Ciphe^, termed here KC-DTR, was developed by the Centre d'Immunophénomique, Marseille, France.

### KC-DTR targeting vector

A genomic fragment encompassing exons 5–7 of the *Clec4F* gene was isolated from a BAC clone of C57BL/6J origin (clone no. RP23-84H4). Using ET recombination, an internal ribosome entry site-YFP-2A-hDTR-loxP-Cre-neoR-loxP cassette was introduced in the 3′ untranslated region of the *Clec4F* gene, downstream of the stop codon. Finally, the targeting construct was abutted to a DTA selection cassette and linearized.

### Isolation of recombinant embryonic stem cell clones

JM8.F6 C57BL/6N ES cells were electroporated with the linearized KC-DTR targeting vector. After selection in G418, ES cell clones were screened for proper homologous recombination by Southern blot. When tested on *Drdl*-digested genomic DNA, a probe used to identify proper recombination events hybridized to a 12.8-kb wild-type fragment. A neomycin-specific probe was used to ensure that adventitious non-homologous recombination events had not occurred in the selected ES clones.

### Production of knock-in mice

Properly recombined ES cells were injected into Friend Virus B (FVB) blastocysts. Germline transmission led to the self-excision of the loxP-Cre-NeoR-loxP cassette in male germinal cells. KC-DTR mice were identified by PCR of tail DNA. In brief, tails were digested overnight with proteinase K (500 μg ml^−1^) at 56 °C. DNA was precipitated by isopropanol, washed in ethanol and resuspended in TER buffer (Tris-HCl 10 mM, EDTA 1 mM, RNase H 20 μg ml^−1^). PCR were performed using a mixture of the three following primers: forward WT primer: 5′- *TCCTACCCCTGGGTGTGCAAGAAGT* -3′; forward KC-DTR primer: 5′- *CACAAGCACTGGCCACACCAAACAA* -3′; reverse WT/KC-DTR primer: 5′- *AAGGGAAGGAGGGGACAGTCCATGG* -3′; This trio of primers amplified a 321-bp band in the case of the WT allele and a 730-bp band in the case of the KC-DTR allele.

### Anti-Clec4f Nb generation

A Nb phage library with an estimated 7.3 × 10^7^ clones was generated by using peripheral blood lymphocytes isolated from an alpaca (*Vicugna pacos*) immunized with recombinant mouse Clec4f protein (R&D system) as described^27^. The selected Nbs were subcloned into the pHEN6 expression vector^28^, which fuses a 6 × His tag to the Nb C-terminus, and confirmed for Clec4F specificity via ELISAs and surface plasmon resonance. Nbs were produced in the periplasm of *E. coli* WK6 cells. Nbs against the *β*-lactamase BcII enzyme of Bacillus cereus (BCII10) with same His tag were used as a control Nb^29^.

### ^99m^Tc-Nanobody labelling and pinhole SPECT/μCT analysis

Nbs were labelled with ^99m^Technetium (^99m^Tc) via their His tag, purified and injected intravenously into mice (1.4±0.53 mCi in 100 μl, corresponding to ∼10 microgram Nb) as described^27^. One hour post injection, anaesthetised mice were imaged using μCT (Skyscan 1178; Skyscan) followed by pinhole SPECT (e.cam180; Siemens Medical Solutions) as described previously^27^.

### Isolation of tissue leukocytes

For the isolation of liver leukocytes, livers were isolated from PBS-perfused mice, chopped finely and incubated for 15–20 min with 1 mg ml^−1^ Collagenase A (Sigma) and 10 U ml^−1^ DNase (Roche) in a shaking water bath at 37 °C. For the isolation of lung, brain and spleen leukocytes, lungs, brains and spleens were isolated from PBS-perfused mice finely chopped and incubated for 30 min with 0.2 mg ml^−1^ Liberase TM (Roche) and 10 U ml^−1^ DNase (Roche) in a shaking water bath at 37 °C. Single cell suspensions from brain were then subjected to a 100:40 percoll gradient (Sigma) to isolate leukocytes. Colonic and small intestinal lamina propria leukocytes were isolated as described previously^30^,^31^.

### Generation of BM chimeras

In all, 6–10-week-old CD45.2 or CD45.1 KC-DTR or wild-type mice were anaesthetized by intraperitoneal administration of Ketamine (150 mg kg^−1^) and Xylazine (10 mg kg^−1^). Livers were protected with a 3-cm-thick lead cover before mice were lethally irradiated with 9 Gy. Once recovered from the anaesthesia, mice were reconstituted by intravenous administration of 10 × 10^6^ BM cells from congenic CD45.1, CD45.2 or CD45.1/CD45.2 BM from wild-type mice. Mice were left for at least 5 weeks before assessing chimerism in the blood and liver by flow cytometry.

### DT-mediated depletion

KC-DTR mice were depleted of KCs by a single intraperitoneal administration of 50 or 2 ng human DT (Sigma).

### Adoptive transfer of Ly6C^hi^ monocytes

Ly6C^hi^ monocytes were FACS-purified from the BM of congenic CD45.1^+^ wild-type mice as live CD45^+^CD11b^+^Ly6C^hi^Ly6G^−^CD115^+^ cells and administered intravenously into CD45.2^+^ KC-DTRxCCR2^−/−^ mice, which had received 50 ng DT i.p. 2–4 h before monocyte transfer to deplete endogenous KCs.

### Flow cytometry

Cells (0.5–5 × 10^6^) were stained with appropriate antibodies (Supplementary Table 1) at 4 °C in the dark for 20 min and were analysed with a Fortessa (BD Biosciences) and FlowJo software (TreeStar). KCs were sorted as live-gated CD45^+^Ly6C^−^Ly6G^−^SiglecF^−^F4/80^+^Clec4F^+^CD11b^int^ cells using an ARIA II or ARIA III (BD, Biosciences). The full list of antibodies used can be found in Supplementary Table 1.

### Assessment of proliferation

For the detection of Ki-67 expression, 3–5 × 10^6^ cells from liver homogenates were first stained with fixable viability dye eFluor780 as per manufacturer's instructions (eBioscience), anti-CD16/32 for blockade of Fc receptors and appropriate extracellular markers (Supplementary Table 1) in the dark at 4 °C. Cells were then fixed and permeabilized at 4 °C for 30 min with a FoxP3 staining buffer kit (eBioscience) before being stained with PerCp-Cy5.5 or BV786 labelled anti-Ki67 (561284, BD biosciences) in the dark at 4 °C for 45 min.

### Assessment of phagocytosis

Cells (2–3 × 10^6^) from liver homogenates were incubated with pHRodo *E. coli* bioparticles as per the manufacturer's instructions and were analysed by flow cytometry.

### Electron microscopy

Sorted em-KCs or mo-KCs were seeded in 24-well plates (Nunc; CellSeed Inc. Labware) and 2 h later, the cells were washed with PBS and fixed in 0.15 M cacodylate buffer with 2.5% paraformaldehyde and 2% glutaraldehyde for 2 h and processed and imaged as previously described^6^.

### Microarray

25,000 Em-KCs from WT and KC-DTR mice with and without DT and Mo-KCs from KC-DTR mice at day 15 and day 30 post DT administration were FACS-purified into 500 μl RLT buffer (Qiagen). RNA was isolated using the micro-RNA isolation kit (Qiagen) and sent to the Nucleomics facility, VIB Leuven, Belgium where the microarrays were performed using the GeneChip Mouse Gene 1.0 ST arrays (Affymetrix). Samples were subsequently analysed using R/Bioconductor. All samples passed quality control, and the Robust Multi-array Average procedure was used to normalize data within arrays (probeset summarization, background correction and log2-transformation) and between arrays (quantile normalization). Only probesets that mapped uniquely to one gene were kept, and for each gene, the probeset with the highest expression level was kept. Principle component analysis plots were created using the 15% of genes with the most variable expression.

### Adoptive transfer to neonates

Total 8 × 10^6^ CD45.1^+^ BM cells were injected intra-peritoneally in 50 μl PBS once to 0–7-day-old CD45.2 WT pups. Mice were sacrificed 8–12 weeks later and the CD45.1 donor-derived cells identified.

### Statistical analysis

Groups were compared with Two-way Student's *t*-test and multiple-group comparisons were performed using one or two way analysis of variance (ANOVA) followed by a Bonferroni post-test with Prism Software (GraphPad Software). Samples were assumed to be normally distributed with similar variance between groups. No randomization was used to determine experimental groups and no blinding of the investigator was performed. Group sizes were determined on the basis of previous experience. No data were excluded from the analyses.

## Additional information

**Accession codes:** All microarray data have been deposited at the National Center for Biotechnology Information Gene Expression Omnibus public database (http://www.ncbi.nlm.nih.gov/geo/) under accession number GSE75225.

**How to cite this article:** Scott, C. L. *et al*. Bone marrow-derived monocytes give rise to self-renewing and fully differentiated Kupffer cells. *Nat. Commun.* 7:10321 doi: 10.1038/ncomms10321 (2016).

## Supplementary Material

Supplementary Information Supplementary Figures 1-5 and Supplementary Table 1
