# Supplementary material for: Bone marrow-derived monocytes give rise to self-renewing and fully differentiated Kupffer cells
Source: Nat Commun. 2016 Jan 27;7:10321. doi: 10.1038/ncomms10321 (PMC4737801; doi:10.1038/ncomms10321)
Supplement: Supplementary Information — Supplementary Figures 1-5 and Supplementary Table 1 [file ncomms10321-s1.pdf]

Supplementary Information:

Supplementary Figures:

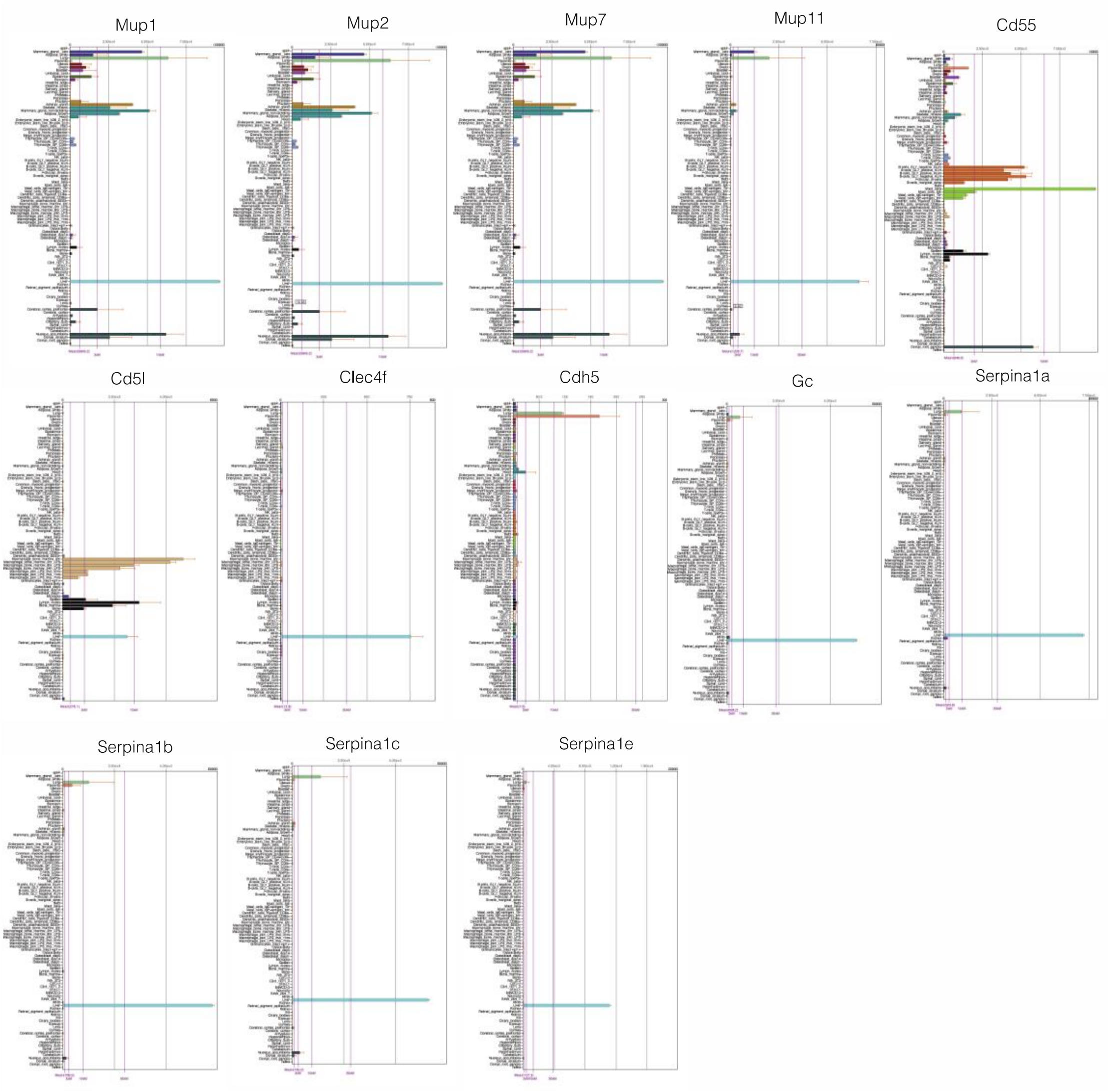

Supplementary Figure 1: Tissue-wide expression of KC-specific genes.
Graphs from BioGPS platform showing tissue-wide expression of indicated KC-specific genes identified from microarray analysis.

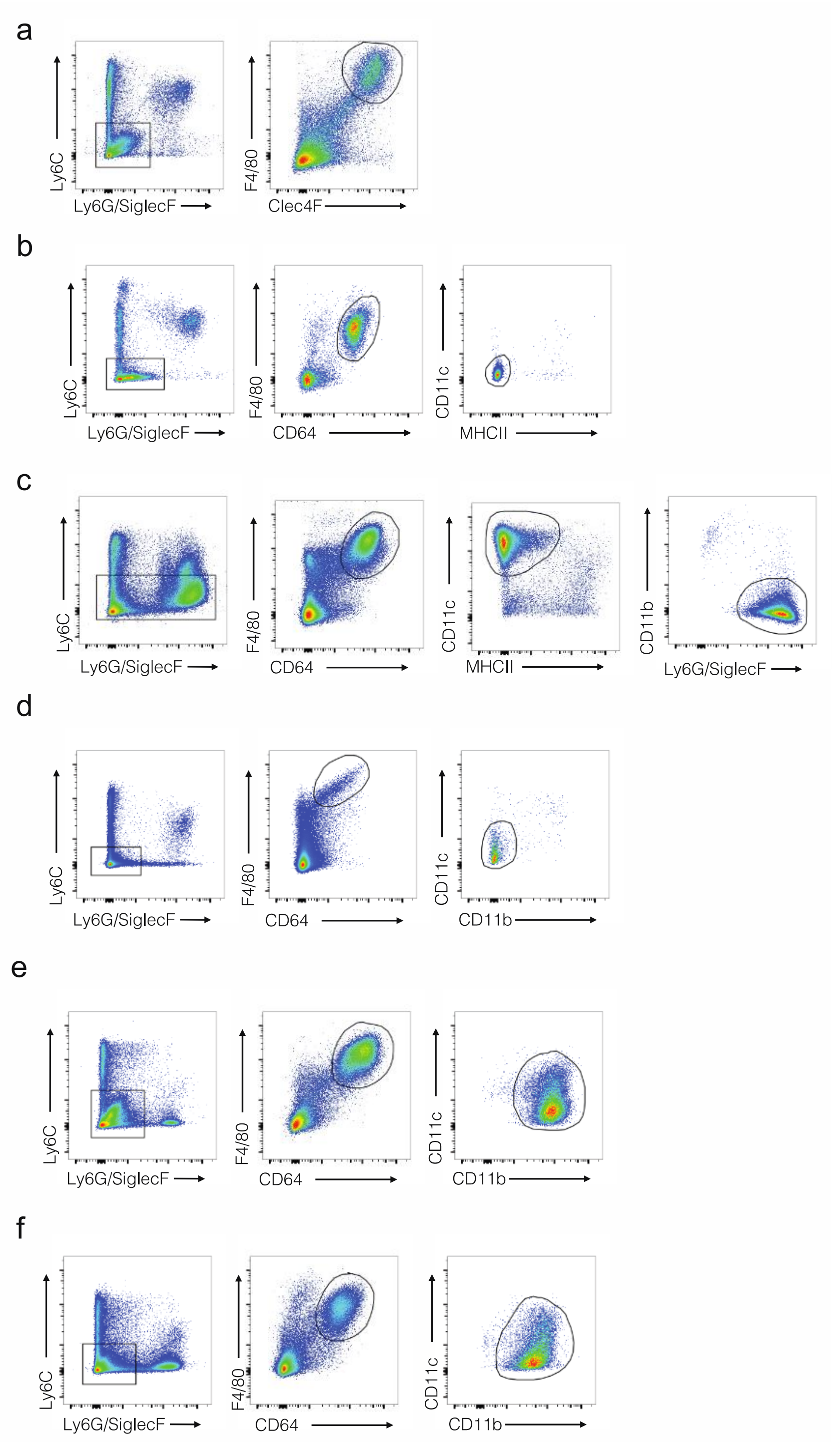

Supplementary Figure 2: Gating strategies for tissue resident mϕ populations. **(a-f)** Representative FACS plot showing identification of (a) KCs, (b) Microglia, (c) Alveolar mϕs, (d) Red pulp mϕs, (e) Colonic LP mϕs and (f) SI LP mϕs from total live $CD45^+$ single cells.

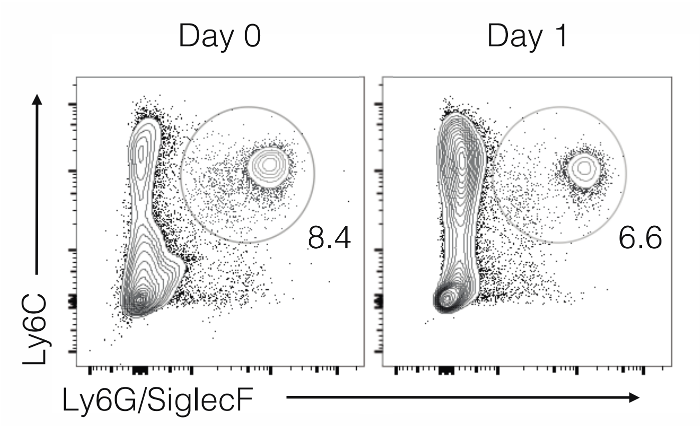

Supplementary Figure 3: Administration of DT does not induce liver inflammation

Ly6C and Ly6G/SiglecF expression in KC-DTR mice 1 day post administration of DT (Day 1) compared with untreated control (Day 0). Data are representative of 3 experiments. n=17(control) or 9(+DT).

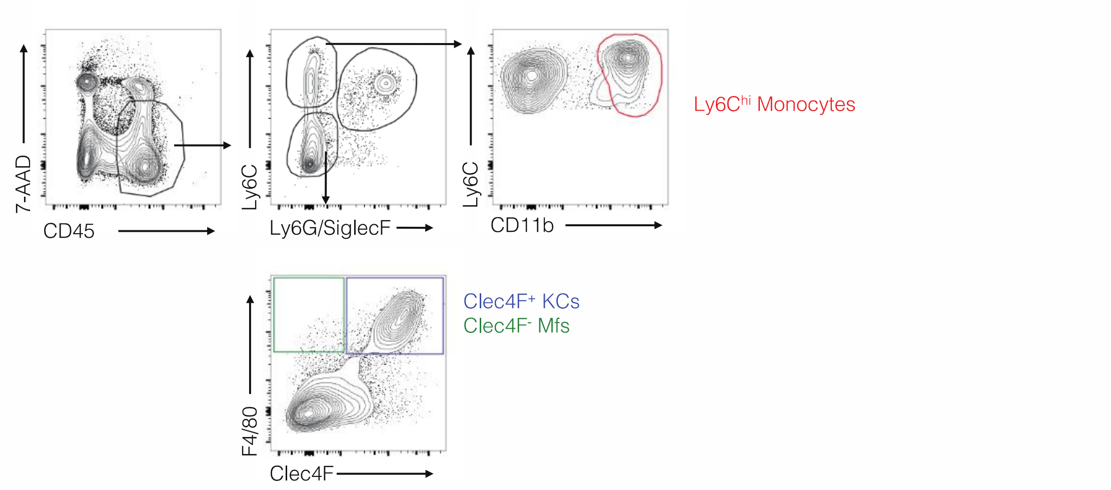

Supplementary Figure 4: Gating strategies for liver Clec4F^{+} KCs, Clec4F^{-} Mϕs and Ly6C^{hi} monocytes.

Representative FACS plot showing identification of Clec4F^{+} KCs, Clec4F^{-} mϕs and Ly6C^{hi} monocytes in the liver from total single cells.

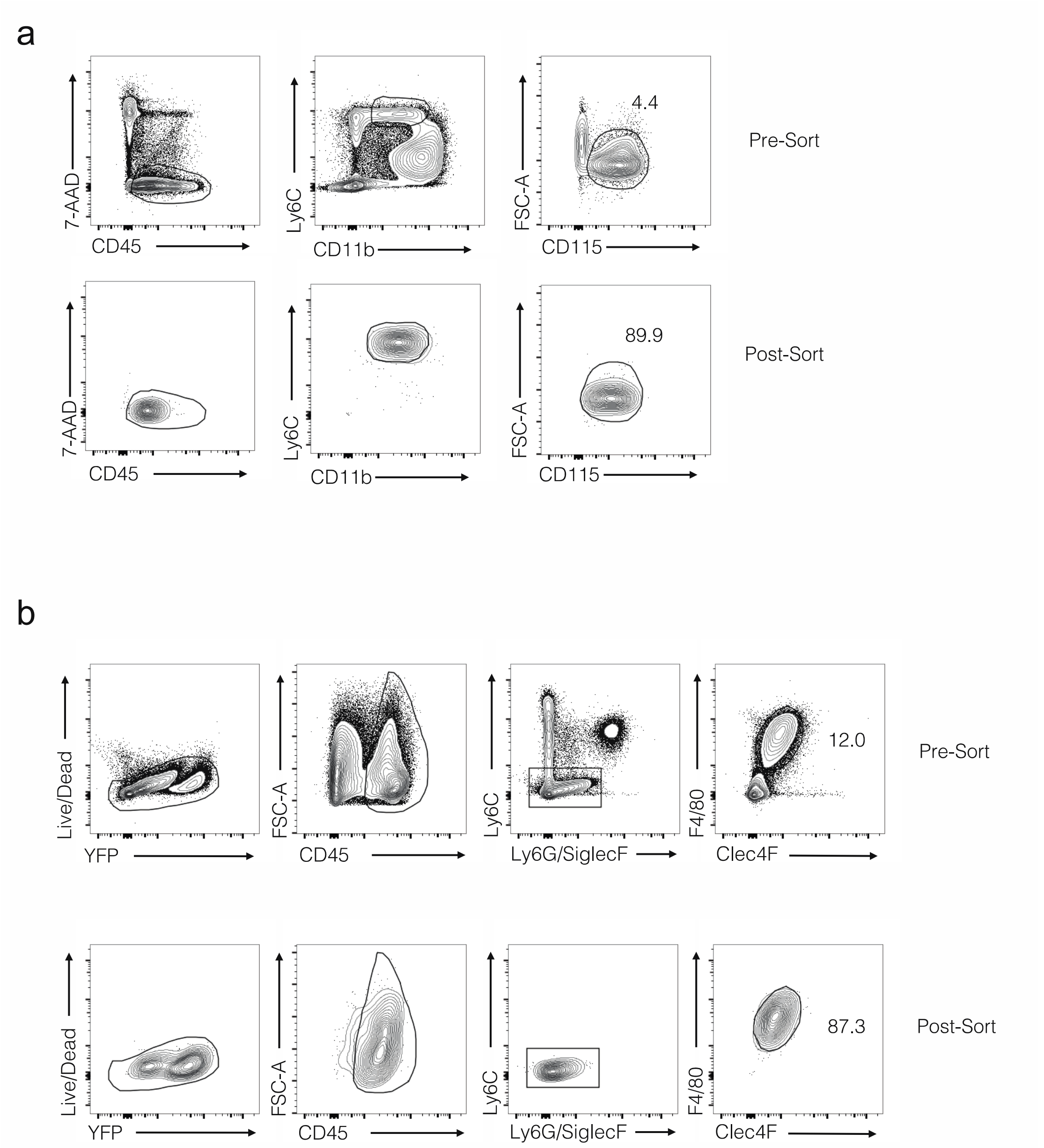

Supplementary Figure 5: Sorting Strategies and purities.
(a) Representative FACS plots showing sorting strategy and purity for BM Ly6C^{hi} monocytes. Plots are pre-gated on single cells. Numbers represent % of total single cells. **(b)** Representative FACS plots showing sorting strategy and purity for em-KCs and mo-KCs for microarray analysis. Plots are pre-gated on single cells and numbers represent % of total single cells.

Supplementary Table 1: Antibodies used

Antibody	Clone	Source	Dilution	Fluorochrome
CD11b	M1/70	Biolegend	1:500	PECy7
CD11c	N418	BD Biosciences	1:400	PECy7
CD115	AFS98	eBioscience	1:100	PE
CD45	30-F11	BD Biosciences	1:200	AF700
CD45.1	A20	BD Biosciences	1:200	PE
CD45.2	104	BD Biosciences	1:200	PerCP-Cy5.5
CD64	X54-5/7.1	BD Biosciences	1:200	AF647
Clec4F	Polyclonal	R&D systems	1:100	-
F4/80	BM8	BD Biosciences	1:200	Biotin
Ki-67	B56	BD Biosciences	1:100	BV786
Ki-67	B56	BD Biosciences	1:100	PerCP-eFluor710
Ly6C	HK1.4	eBioscience	1:500	eFluor450
Ly6G	1A8	BD Biosciences	1:800	PE
MHCII	M5/114.15.2	eBioscience	1:500	APC-eFluor780
SiglecF	E50-2440	BD Biosciences	1:800	PE
Streptavidin	-	Invitrogen	1:400	QDot605
Anti-goat IgG	-	Invitrogen	1:1000	AF647
